# Global caregiver perspectives on COVID-19 immunization in childhood cancer: A qualitative study

**DOI:** 10.3389/fpubh.2023.1004263

**Published:** 2023-03-07

**Authors:** Allison Silverstein, Julia M. Gumy, Jessica Bate, Erica C. Kaye

**Affiliations:** ^1^Division of Quality of Life and Palliative Care, St. Jude Children's Research Hospital, Memphis, TN, United States; ^2^Division of Pediatric Palliative Care, University of Tennessee Health Science Center, Memphis, TN, United States; ^3^School for Policy Studies, University of Bristol, Bristol, United Kingdom; ^4^Department of Paediatric Oncology, Southampton Children's Hospital, Southampton, United Kingdom

**Keywords:** pediatric oncology, SARS-CoV-2, immunization, global health, public health

## Abstract

**Introduction:**

SARS-CoV-2 has led to an unprecedented pandemic where vulnerable populations, such as those with childhood cancer, face increased risk of morbidity and mortality. COVID-19 vaccines are a critical intervention to control the pandemic and ensure patient safety. This study explores global caregiver's perspectives related to COVID-19 immunization in the context of pediatric cancer management.

**Methods:**

A mixed methods survey was developed based on consensus questions with iterative feedback from global medical professional and caregiver groups and distributed globally to caregivers of childhood cancer via electronic and paper routes. We present qualitative findings through inductive content analysis of caregiver free-text responses.

**Results:**

A total of 184 participants provided qualitative responses, 29.3% of total survey respondents, with a total of 271 codes applied. Codes focused on themes related to safety and effectiveness (*n* = 95, 35.1%), logistics (*n* = 69, 25.5%), statements supporting or opposing vaccination (*n* = 55, 20.3%), and statements discussing the limited availability of information (*n* = 31, 11.4%). Within the theme of safety and effectiveness, safety itself was the most commonly used code (*n* = 66, 24.4% of total segments and 69.5% of safety and effectiveness codes), followed by risks versus benefits (*n* = 18, 18.9% of safety and effectiveness codes) and efficacy (*n* = 11, 11.6%).

**Discussion:**

This study provides insights to guide healthcare professionals and caregiver peers in supporting families during the complex decision-making process for COVID-19 vaccination. These findings highlight the multidimensionality of concerns and considerations of caregivers of children with cancer regarding COVID-19 vaccination and suggest that certain perspectives transcend borders and cultures.

## Introduction

Vaccination decision-making has challenged healthcare professionals for decades, with vaccine hesitancy remaining a significant threat to global public health in the 21st century (1, 2). With the emergence of the SARS-CoV-2 pandemic and relatively recent approvals for vaccines for pediatric populations, global public concerns around vaccine safety and value for children have further intensified in recent months (3–6). These growing concerns not only threaten vaccine rates for community protection over time, but also more immediately place vulnerable individuals at increased risk.

The virulence of SARS-CoV-2, resulting in staggering morbidity and mortality worldwide from the disease known as COVID-19, has underscored the urgent need to explore and better understand roots and drivers behind vaccine decision-making, particularly within vulnerable pediatric subpopulations. While healthy children and adolescents infected by SARS-CoV-2 generally experience milder illness than adults (7), the Global Registry of COVID-19 in Childhood Cancer revealed that children and adolescents with cancer are more likely to develop severe or critical illness when exposed to SARS-CoV-2. Specifically, one in five patients developed severe or critical illness and ~4% died, well above the projected statistics for healthy children (8). Additional reviews with global perspectives have emphasized this increased risk amongst patients with childhood cancer (9, 10).

While numerous studies have investigated attitudes and perceptions surrounding COVID-19 vaccination in adults (11–17), fewer studies have examined parental considerations for COVID-19 vaccination of children in the setting of recent authorization of a pediatric vaccine (3, 5, 6, 18–26). To our knowledge, one prior study has explored vaccine willingness and hesitancy in the context of pediatric cancer, targeting the views of U.S.-based caregivers of childhood cancer survivors; within this cohort, 29% of caregivers expressed vaccine hesitancy, and confidence in COVID-19 vaccination and its value for childhood cancer survivors emerged as a prominent theme (27).

The 2013 World Health Organization (WHO) Strategic Advisory Group of Experts on Immunization (SAGE) Vaccine Hesitancy Working Group recognized vaccine decision-making as a complex and dynamic process where certain factors may be more important in specific contexts or during certain experiences (28). Currently, the perspectives, values, and concerns of caregivers about COVID-19 vaccination for children with cancer globally remain poorly understood. Understanding the views of pediatric cancer caregivers on COVID-19 immunization is important to enable healthcare professionals to better support families and provide anticipatory guidance on vaccine administration.

To address this gap in knowledge, a Vaccine Working Group collaboration between the International Society of Paediatric Oncology (SIOP) and St. Jude Children's Research Hospital (SJCRH) was formed with the goal of better understanding COVID-19 vaccine decision-making related to the care of children with cancer. In this paper, we present qualitative findings from a global assessment of caregiver perspectives related to COVID-19 vaccination in the context of pediatric cancer management.

## Materials and methods

### Survey tool development

A COVID-19 Vaccine Working Group on Pediatric Oncology was established in March 2021 to answer and investigate COVID-19 vaccine questions. Twelve members consisting of oncologists, infectious disease physicians, and nurses were selected to represent various regions around the world. Working group members nominated parent representatives to contribute to the project from their own country including the United Kingdom, the United States, Canada, Philippines, Indonesia, India, and Ghana. These parents established the Parent/Carer Advisory Group, comprising nine individuals representing patients with cancer and their families from various global regions (29).

A mixed methods survey was developed, guided by content from a professional statement by the COVID-19 and Childhood Cancer Vaccine Working Group collaboration between SIOP and SJCRH (30). The initial consensus questions were derived from global professional healthcare organizations and narrowed via a modified Delphi method amongst the Vaccine Working Group members, with a total of three voting sessions to reach consensus. The initial consensus questions were reviewed by the Parent/Carer Advisory Group, and members of the Advisory Group contributed or revised question items as needed to strengthen face and content validity; the survey underwent iterative stages of feedback with collaborative Advisory Group review to yield the final survey. The survey was piloted with a small group of parents with experience in childhood cancer to test face and content validity of the question items.

The final survey contained three background questions, 19 quantitative Likert scale questions, and a summative open-ended question asking participants to share their questions and perspectives about administration of the COVID-19 vaccine in children with cancer; the survey instrument is presented in Supplementary Table 1. The background or demographic questions focused on country of residence, type of childhood cancer, and timing of the child's cancer experience.

### Eligibility criteria, recruitment, enrollment, and data collection

Any parents or primary caregivers of those with childhood cancer were eligible for participation. Each member of the Parent/Carer Advisory Group disseminated the survey to respondents in their own country primarily via social media, online forums, and email distribution. The Working Group members also disseminated the survey to caregivers in each of their countries. The survey was primarily distributed online via SurveyMonkey. A small proportion of respondents (i.e., those from South Africa and Ghana) were approached with paper forms due to limited WiFi in the clinic space where surveys were distributed; responses were then entered manually into the electronic database. The survey was translated into Spanish for dissemination in Spanish-speaking countries; otherwise, an English version was distributed. The survey was disseminated between April and May 2021, remaining open for 4 weeks. Sampling utilized convenience and snowball techniques, with an emphasis on targeting existing pediatric cancer caregiver forums including the international Momcology email distribution listserv and other country-specific online and social media pediatric cancer caregiver communities. Following collection of data via SurveyMonkey and paper surveys, a de-identified CSV file was produced, and a targeted file comprising demographic characteristics and open ended item responses was uploaded to MAXQDA, a qualitative and mixed methods data analysis software system.

The study was classified as informational by SJCRH and exempt from Institutional Review Board approval. The involvement of patients and public advisors was also not deemed subject to ethical approval by the U.K. National Research Ethics Services. Following a brief introduction to the aim, respondents provided informed consent prior to survey completion by answering “yes” to the first question explaining inclusion criteria and that no identifiable information would be collected.

### Data analysis

This article presents findings from qualitative analysis of the summative open-ended question; analysis focused on responses to a single free text qualitative question, and all those who provided free text responses were included. Any free text response was considered to be a complete unit of response. We describe study methods and findings following the COREQ (Consolidated Criteria for Reporting Qualitative Research) checklist (Supplementary Table 2) (31). Inductive content analysis was conducted across free-text responses by researchers representing three distinct perspectives: (1) a pediatrician with global health training and expertise (A.S.), (2) a parent of a child with cancer with population health research expertise (J.G.), and (3) a pediatric oncologist with qualitative research expertise (E.K.) (Supplementary Table 3) (32).

Research analysists (A.S., J.G., E.K.) reviewed transcripts in depth and conducted memo-writing to begin identifying concepts and patterns. Through this process, an inductive codebook was developed and refined iteratively until no further concepts were identified and saturation was achieved. Code definitions and examples were pilot-tested (A.S., J.G.) across complex responses to identify areas of variance, with minor modifications to language and content made as needed to ensure consistency in code application across transcripts (A.S., J.G., E.K.). The final codebook comprised six broad categories which included a total of 17 codes and two embedded subcodes (Table 1).

**Table 1:** Codebook.

**Code**	**Definition**
**Logistics**	
Who	**Who should get the vaccine?**• Includes any questions or statements about who should receive the vaccine and/or in what order (prioritization/triaging). • *(1) For the cancer patient themselves (e.g., immunocompromised, stem cell recipient)* ° *Questions specific to stratifying prioritization within this group* °*What age should start getting the vaccine?* • *(2) For household members/siblings* • *(3) For other close family and friends*
When	**When is best to vaccinate?** • Includes any questions or statements about timing to receive the vaccine. • *Questions about recommendations for those with active cancer, those who finished treatment, or long-term survivors* • *Is it ideal to give before, during, or after finish chemo? Should we pause chemotherapy?* • *What about those who have had delayed/no immunizations due to cancer/treatment? (e.g., bone marrow transplant patients)*
How	**How is best to vaccinate?** • Includes any questions or statements about the best administration regimen to promote efficacy or immunity. • *What is optimal timing between doses?* • *At what frequency should the vaccine be given?* • *Is there need to re-vaccinate or give a booster?* • *Can we do antibody testing/titers to avoid a false sense of security?*
Where	**Where is best to vaccinate?** • Includes any questions or statements about the location that is best to vaccinate. • *Hospital, homecare, doctor's office*
Which	**Which is the best vaccine for this population?** • Includes any questions or statements about which vaccine option is best for the population. Also includes questions about protectiveness against variants.
Contraindications	**Contraindications for vulnerable sub-populations**. • Includes any questions or statements about which populations of patients may be at higher risk of side effects, decreased efficacy, or other undesirable issues. • *Trisomy 21; single kidney; T-ALL; radiation; allogeneic transplant; if genetic predisposition; if allergy (e.g., to peg-asparaginase)*
**Safety and effectiveness**	
Safety *subcodes:* Safety _Acute Safety _Chronic	**Safety related to oncology or general health**. • Includes any questions or statements specific to potential side effects of the vaccine. Includes both short- and long-term potential effects of vaccine. • **Acute** • *Potential to slow healing process after chemo or leads to challenges if during chemotherapy?* • *Any interactions with immunosuppressants/other cancer-directed therapies?* • *Potential to disrupt immune system's ability to fight off cancer post vaccine?* • *Potential for tumor growth or activation of graft vs. host disease (GVHD)?* • *Potential to interfere with scan results?* • *Any flu-like symptoms, fatigue, swelling at site, inflammatory response, respiratory issues, blood clots?* • *Okay in those breastfeeding?* • *Potential to shed from others vaccinated?* • **Chronic** • *Potential to trigger relapse/growth?* • *Risk for developing secondary cancer?* • *In those off treatment but with long-term health conditions from treatment?* • *Potential for infertility?* • *Any potential to disrupt child development?*
Risk vs. benefits = RvB	**Risk vs. benefits of vaccine in comparison to risk of getting COVID-19**. • Includes any questions or statements specific to risk vs. benefits of the vaccine specifically in comparison to risk of a child getting COVID-19. • *Feelings that vaccine potentially carries greater risk than virus (e.g., child already recovered from COVID without issues)* • *Question of short vs. long term effects in children with cancer, risk for severe reaction to vaccine vs. severe illness with COVID* • *Are children with history of cancer at higher risk of poor outcomes with COVID?* • “*Since kids rarely get serious COVID, why is this needed?”*
Efficacy	**Efficacy in sub-populations**. • Includes any questions or statements about ability of specific populations of patients to mount protective response to vaccine. • *If on chemotherapy? With low T cells? Low IgG? On neulasta? Low blood counts? Following CAR-T?*
**Overall frustrations**	
Frustration	**Frustration toward those not getting the vaccine**. • *Lack of herd immunity placing children with cancer at risk*
Access	**Worry or anger regarding difficulty with access or lack of access to vaccine**.
**Availability of information**	
Guidance	**Wanting guidance**. • Includes any questions or statements seeking guidance or advice in making decision to vaccinate or not vaccinate child. • *Wanting provider advice regarding choosing between types of vaccines* • *Planning to ask doctor for input/recommendation*
Limited information = limited	**Uncertainty and lack of clarity amongst studies about effects of COVID and the vaccine and need for more research**. • *Wanting to know more information, wanting transparency* • *Worries about frequently changing information* • *Expressions that need to include children in research; children with cancer; minorities; double blind study; animal models; long-term studies*
**Expressions pro/con**	
Refusal	**Refusal**. • Includes any statements or thoughts against the vaccine. Includes statements of intent not to get the vaccine. Nuanced differences from hesitancy. • *Children already suffering enough, don't need other chemicals/toxins/metal* • *Not enough known, no longevity studies* • *Feelings that masks are enough* • *Vaccine is experimental and only approved for emergency use* • *Not willing to do another “experimental” therapy, expressions of anger* • *Other vaccines not offered during treatment, COVID vaccine shouldn't be given either* • *Comments that child already got COVID and has natural antibodies* • *Do not support in children, with or without cancer; against vaccine in self* • *Fear that children with cancer not as strong secondary to chemotherapy* • *Vaccine is a scam; conspiracy; etc*.
Hesitancy	**Reluctance or skepticism/doubt about vaccine**. • Includes any statements or questions that are not made in a clear pro or con mindset. • *Don't believe children are affected by COVID or only mildly if so* • *Concern about vaccine ingredients* • *Not wanting the vaccine to be mandated*
Favor	**In favor**. • Includes any statements or thoughts in favor of the vaccine. Includes statements sharing having received the vaccine or intent to. • *Examples of self or child with cancer having received vaccine* • *Agree with prioritization as these children already have long-term treatment side effects to manage*
**Other**	
Feedback	• Feedback on overall study, design, content, importance, both for/against

The codebook was applied across all responses (A.S., J.G.) with data organized in MAXQDA. The research team met at regular intervals to review findings and reconcile variances, with third-party adjudication (E.K.) to achieve consensus. For responses that met criteria for multiple codes, responses were dual-coded to capture diversity and nuance within perspectives. Following finalization of coding, the team reviewed codes to identify patterns and generate themes (33). Once patterns were established, the team conducted quantitative analyses to describe frequencies of responses. Available demographics (e.g., respondent country's World Bank Income Group and WHO Region, type of childhood cancer, and timing of child's cancer experience) were evaluated for differences between those who responded to the qualitative question compared to those who did not and the entire cohort.

## Results

Of the 627 total survey participants from 22 countries, a total of 184 persons (29.3%) provided free-text comments. Broad patient characteristics of those who responded to the open-ended question were similar to those who opted not to respond to the question (Table 2).

**Table 2:** Survey participant characteristics.

	**Qualitative respondents (*n* = 184)**	**No qualitative response (*n* = 443)**	**Overall surveyed (*n* = 627)**
**World Bank income group**			
Low middle income	8 (4.4)	18 (4.1%)	26 (4.2%)
Upper middle income	15 (8.2%)	26 (5.9%)	41 (6.5%)
High income	161 (87.5%)	398 (89.8%)	559 (89.2%)
Unknown	0 (0%)	1 (0.2%)	1 (0.2%)
**WHO region**			
African region	18 (9.8%)	28 (6.3%)	46 (7.3%)
European region	15 (8.2%)	47 (10.6%)	62 (9.9%)
Region of the Americas	145 (78.8%)	348 (78.6%)	493 (78.6%)
Southeast Asian region	4 (2.2%)	14 (3.2%)	18 (2.9%)
Western Pacific region	2 (1.1%)	5 (1.1%)	7 (1.1%)
Unknown	0 (0%)	1 (0.2%)	1 (0.2%)
**Type of cancer**			
Leukemia (e.g., ALL, AML)	91 (49.5%)	234 (52.8%)	325 (51.8%)
Lymphoma (e.g., B-NHL, Hodgkins disease)	17 (9.2%)	41 (9.3%)	58 (9.3%)
Brain or spinal tumor (e.g., ependymoma, medulloblastoma)	23 (12.5%)	58 (13.1%)	81 (12.9%)
Solid tumor outside the brain (e.g., Wilms, neuroblastoma, sarcoma)	49 (26.6%)	106 (23.9%)	155 (24.7%)
Other	1 (0.5%)	2 (0.5%)	3 (0.5%)
Unknown	0 (0%)	2 (0.5%)	2 (0.3%)
**Timing of cancer experience**			
Within last 12 months	19 (10.3%)	60 (13.5%)	79 (12.6%)
Between 1 and 3 years ago	60 (32.6%)	159 (35.9%)	219 (34.9%)
Between 3 and 5 years ago	52 (28.3%)	98 (22.1%)	150 (23.9%)
More than 5 years ago	53 (28.8%)	126 (28.4%)	179 (28.6%)

ALL, acute lymphocytic leukemia; AML, acute myeloid leukemia; B-NHL, B-cell non-Hodgkin lymphoma.

**Figure 1:**
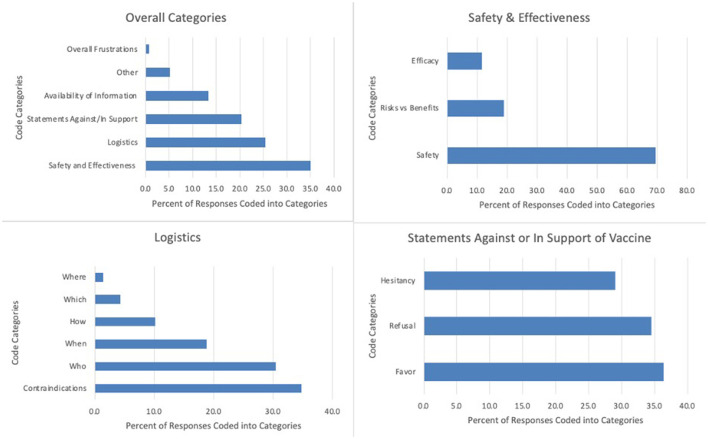
Frequencies of codes within each thematic domain.

Across the transcript of free-text responses from 184 respondents, a total of 271 codes were applied. Approximately one-third of codes (*n* = 95, 35.1%) were related to safety and effectiveness, one-quarter (*n* = 69, 25.5%) related to logistics, and one-fifth (*n* = 55, 20.3%) related to statements in support of or against vaccination. Other emerging concepts included availability of information (*n* = 36, 13.3%) and overall frustrations (*n* = 2, 0.7%). The remaining codes referenced survey feedback or “other” comments not related to identified themes (*n* = 14, 5.2%). Supporting quotations for each of the themes are presented in Table 3. Frequencies of codes within each thematic domain are shown in Figure 1. Results from these analyses aligned with quantitative themes identified by Principal Component Analysis (34).

**Table 3:** Supporting quotations.

**Code**	**Examples/supporting quotations**
**Logistics**	
Who should get the vaccine?	• “My son is 5 and is undergoing treatment for ALL so I am not sure if/when he can get the vaccine during treatment.” • “How do we prioritize immunocompromised cancer patients and stem cell transplant recipients to ensure that they get the vaccine ASAP when approved?” • “Should siblings and/or close family and friends also receive the vaccination?”
When is best to vaccinate?	• “What will be the recommended timeframe to receive the vaccine for children off treatment?” • “I think that a vaccine for children before chemotherapy is critical.” • “I want the children to complete chemotherapy before the vaccine is administered.” • “Should the COVID vaccine be given when children repeat their childhood immunisations post treatment or does there need to be a delay?”
How is best to vaccinate?	• “…An important question for lymphoma kids is what interval there needs to be between doses to achieve maximum immunity.” • “How often should children undergoing treatment be given COVID vaccine boosters?” • “Will there be follow up to check for titers after the COVID vaccine? I don't want a false sense of security that my child is protected from COVID when indeed he may not be.”
Where is best to vaccinate?	• “What is the best way to vaccinate these children and their caregivers (In hospital clinic? Homecare visit? Family doctor's office?)?”
Which is the best vaccine?	• “As the confusion around types of vaccines…I would be very interested to know which type of vaccine would be recommended for children with cancer—if there was a distinction.” • “Also, which vaccine is recommended for immunocompromised.” • “Is it safe for her to have one? What is riskier, that or? Blood clot from the other two?”
Contraindications	• “Is it true that a patient who has had an allergic reaction to pegasparaganase cannot have the vaccine?” • “If children who had a kidney removed due to wilms tumor can receive the vaccine? Is it safe for kids with single kidney?” • “If a child on Neulasta can they still get the vaccine?” • “Can it be given while blood counts are very low due to chemotherapy.”
**Safety and effectiveness**	
Safety	• “Safety and protection are my biggest concerns” • “I would like to know if it is safe for them to take the vaccine…Will it have side effects.” • “My son has completed his treatment but I am still concerned whether the vaccine for him as well as my other son for that matter is safe and the best option with no real evidence for the safety of children.” • “Vaccine safety and side effects (which may be different from children that haven't had cancer) are extremely important. My child is no longer on active treatment and hasn't been for years but she has a bunch of long term effects from surgery and treatment. Will this vaccine have any impact on them?”
Acute safety	• “Will the vaccine interfere in results of scans?” • “Any risks to kids who are recently off treatment and are just rebuilding their immune systems?” • “Is it safe for a mom breastfeeding her cancer child who is off treatment to get the vaccine? Is it safe for a mom breastfeeding her cancer child who is receiving chemo to get the vaccine?” • “Many adults who have received the vaccine experience flu-like symptoms, to varying degrees of severity. Would children with cancer be more likely to experience worse flu-like symptoms as a natural reaction to the vaccine?”
Chronic safety	• “For kids who have had radiation-would the vaccine put them at any higher risk of developing another cancer later? These kids are already so much more at risk for secondary cancers as they age—how would this vaccine impact those risks?” • “What are the long term effects of the vaccine? With children that have been treated with radiation and chemotherapy there are often multiple long term effects. How will the long term effects of the vaccine affect those?” • “How will it affect them long term. In general, what does the vaccine do for fertility...” • “There are so many unknowns at this point about long term side effects.”
Risk vs. benefits	• “I'd like to know the benefits outweigh the risks. My son has had COVID and it really didn't effect him so I'd be reluctant to give him a vaccine as the risks of the vaccine would be potentially more than getting the virus.” • “Need for understanding of risk of COVID vs. risk of vaccine for cancer kids.” • “What is the relative likelihood of a child with cancer having a severe reaction to vaccine vs. severe illness with COVID?” • “I don't care how many booster doses I would need, getting the vaccine certainly outweighs the “newness” and “inconvenience”, plus the chance effects of COVID.”
Efficacy	• “How much will chemotherapy intensity affect the vaccine efficacy and are there any objective tests that can prove vaccine efficacy” • “Will the vaccine work on a child whose IgG levels are impaired post-chemotherapy?” • “I want to know…how effective it will be. Will it be less effective since his lymphocyte count is still low.”
**Overall frustrations**	
Frustration	• “It is also difficult convincing grown humans who are healthy to get the vaccine to protect kids like my son. It is frustrating.”
Worry or anger regarding access	• “I cannot believe how hard I had to fight to get the vaccine for my daughter. JCVI you should be ashamed of yourselves!!!”
**Availability of information**	
Wanting guidance	• “And for the children with cancer I will still ask the doctor whether to take vaccine or not” • “Should I confirm with my pediatric oncologist first before taking it.” • “…his Dr. said that he can get it so we have him scheduled for an appointment”
Limited information	• “More studies have to be done on chemotherapy and the vaccine because of the so many long term side effects of chemotherapy” • “There are not nearly enough studies or research to determine what the side effects could be.” • “My daughter will not be receiving the vaccine until more studies have been done.” • “Are children with cancer in any of these studies for COVID vaccine? Our children are different and therefore react differently to this vaccine”
**Statements frankly against or in support of vaccine**	
Refusal	• “It should not be given. Not enough knowns and absolutely zero longevity studies. Our kids suffer enough without added man made chemicals and concoctions.” • “We will not be getting the vaccine nor will any of our children.” • “I will never allow my child to get the COVID-19 vaccine…if childhood cancer really wants to find a cure, they should stop injecting METAL into these poor children…. Sincerely, an angry mother.” • “I don't think any child should get this vaccine. Let alone a child with cancer. NO ONE SHOULD BE RECEIVING THIS VACCINE”
Hesitancy	• “Hope these questions/thoughts offer some helpful insights into the mind of a fully vaccinated parents of a child diagnosed with cancer). Definitely not against the vaccine, just have a lot of unanswered questions.” • “We are questioning whether it is worth getting the vaccine given the increasing number of variants/it may not be effective and comes with a risk of side effects.” • “Why is there cells/DNA from animals and aborted fetuses in the vaccine?”
In favor	• “Research and answers need to happen immediately—pediatric cancer patients have not had the chance to live full lives and if they beat cancer shouldn't then die from COVID? They should have been vaccinated with first group like essential workers instead of last?” • “My 21 year old with leukemia got the vaccine done and did very well with vaccine, he was diagnosed May 2018 and still has 6 months left in treatment” • “Let's start vaccinating!”
**Other**	
Feedback	• “All of the questions above are so important.” • “There should be questions about immunotherapy not just chemotherapy. So many questions about whether vaccine is effective w Car T immunotherapy kids” • “For the responses I answered “not at all” it was because that information feels already available and accessible.” • “It is hard to answer some of these questions based on how they are written” • “I did not answer questions that did not apply to our situations such as children that have already had COVID-19 or have had a bone marrow transplant.”

### Safety and effectiveness

Statements related to safety and effectiveness were most commonly coded (*n* = 95, 35.1%). Within this category, statements asking questions or expressing concerns about safety were the single most used code (*n* = 66, 24.4% of total segments and 69.5% of safety and effectiveness codes). One caregiver asked, “*Vaccine safety and side effects (which may be different from children that haven't had cancer) are extremely important. My child is no longer on active treatment and hasn't been for years but she has a bunch of long term effects from surgery and treatment. Will this vaccine have any impact on them?”* Seven of these 66 coded safety segments were double coded as containing both acute and chronic or acute/chronic and non-specific safety comments. Of safety-specific segments, 20.5% (*n* = 15) specifically addressed acute safety concerns and 37.0% (*n* = 27) addressed chronic safety. Acute safety concerns included comments such as, “*Does the vaccine have the possibility of affecting how well my child's body will be able to fight off her cancer cells?”* while chronic safety included questions such as, “*How will it affect them long term. In general, what does the vaccine do for fertility….”* Some safety codes addressed broad safety concerns, relevant to the general pediatric population, while others were specific to oncologic concerns. The remaining safety and effectiveness codes addressed risks vs. benefits (*n* = 18, 18.9% of safety and effectiveness codes): “*What is the relative likelihood of a child with cancer having a severe reaction to vaccine vs. severe illness with COVID?”;* and efficacy (*n* = 11, 11.6%): “*I want to know…how effective it will be. Will it be less effective since his lymphocyte count is still low?”*

### Logistics

Out of the 69 coded segments related to logistics, 24 (34.8%) were specific to contraindications. One caregiver stated, “*Is it true that a patient who has had an allergic reaction to pegasparaganase [PEG-asparaginase] cannot have the vaccine?”* A similar number of segments (21, 30.4%) focused on who should get the vaccine (“*Should siblings and/or close family and friends also receive the vaccination?”*), while relatively fewer codes (13, 18.8%) centered on optimal timing for vaccination (“*What will be the recommended timeframe to receive the vaccine for children off treatment?”*). Other logistical concerns underscored best practices for children actively receiving cancer-directed therapy (7, 10.1%: “*How often should children undergoing treatment be given COVID vaccine boosters?”*); which vaccine is best (3, 4.3%: “*As the confusion around types of vaccines…I would be very interested to know which type of vaccine would be recommended for children with cancer—if there was a distinction.”*); and where to receive the vaccine [1, 1.4%: “*What is the best way to vaccinate these children and their caregivers (In hospital clinic? Homecare visit? Family doctor's office?)?”*].

### Statements against or in support of vacciation

Twenty (36.4%) of the 55 broad category statements were in favor of the vaccine, 19 (34.5%) refusing the vaccine, and 16 (29.1%) reflecting hesitation about the vaccine. Statements in favor included, “*Let's start vaccinating!”* Conversely, refusal statements expressed, “*I will never allow my child to get the COVID-19 vaccine.”*

### Availability of information

A total of 31 statements (11.4% of all coded segments) discussed the limited availability of information about the vaccine, with one parent commenting, “*My daughter will not be receiving the vaccine until more studies have been done.”* Other comments reflected wishes for guidance in their decision-making process given dearth of available information, such as, “*Should I confirm with my pediatric oncologist first before taking it.”*

### Overall frustrations

One caregiver discussed frustrations toward those not getting the vaccine: “*It is also difficult convincing grown humans who are healthy to get the vaccine to protect kids like my son. It is frustrating.”* Another expressed frustrations around difficulty with access to the vaccine: “*I cannot believe how hard I had to fight to get the vaccine for my daughter….”*

## Discussion

This study explores qualitative responses from a global assessment of caregiver perspectives on COVID-19 vaccination in childhood cancer, with the goal of gaining insights to guide healthcare professionals in supporting families during their complex decision-making process. As the first global study specific to childhood cancer to investigate COVID-19 vaccine views, we identified distinct themes with nearly three-quarters of caregiver comments focused on safety and effectiveness, logistics, and limited information to guide decision-making.

Although attitudes specific to COVID-19 vaccination are complex and multifactorial, thematic patterns appear to transcend borders and cultures. Studies from various countries consistently show that safety, effectiveness, and limited information are significant drivers of COVID-19 vaccination in Brazil (3), China (5, 25), Saudi Arabia (4), Turkey (22, 23), the United States (35, 36), and other countries. Our findings corroborate vaccine safety and effectiveness as a primary consideration across multiple countries, comprising over one-third of narrative content. More than one in ten caregivers commented on the availability of information, primarily related to how perceived deficits in knowledge adversely impacted their decision-making.

Among the 184 qualitative responses, a total of 271 codes were applied. This breadth of inductive coding underscores the multidimensionality of perspectives, where many caregivers considered multiple factors of vaccination and were not focused on one aspect of care. This highlights the complexity of caregivers' views and the need for healthcare providers to discuss a variety of considerations. Importantly, explored dimensions may be interrelated, and caregiver questions should be explored with awareness of how they may connect to other questions to encourage vaccine uptake.

With respect to safety, more caregivers reflected about chronic or long-term side effects compared to acute side effects. This may reflect uncertainty and fear related to limited knowledge about long-term side effects given the novelty of the COVID-19 vaccine. Caregivers already face uncertainty and fear about long-term impacts of cancer and cancer therapy on their child's future, which may exacerbate worries about any additional long-term vaccine effects. Additionally, we hypothesize that caregivers also may have concerns about long-term effects as a result of their prior or ongoing experiences with long-term effects from cancer treatment, sensitizing them toward these risks. Healthcare professionals should recognize these fears with compassion, acknowledge when data are limited, and anchor discussion and recommendations about vaccines in available information to address specific concerns. While the COVID-19 vaccine itself is relatively new, the science underpinning its development and the efficacy and safety of vaccination programs are supported by decades of extensive testing and expert guidance (37, 38). Public health strategies should focus on existing information to address myths or fears related to long-term effects.

Additionally, evidence suggests that perceived risk for COVID-19 disease in children informs parental decision-making (26, 27, 35, 39, 40). Our findings corroborate this phenomenon, with some caregivers questioning or asserting that children are unlikely to transmit or develop serious illness from COVID-19 while others believed that children with cancer face increased risk. We encourage healthcare professionals to explore upfront caregiver beliefs about COVID-19 risks to children prior to offering recommendations about vaccination. When caregivers think risk is negligible, early discussion around known risks of COVID-19 may lay a better foundation upon which to build future recommendations.

Caregivers also repeatedly expressed concerns about limited information and frustrations that data for children are often lagging. These data build upon existing research in which parents express a need for better evidence and transparency about vaccine development, efficacy, and safety (39). In pediatric cancer as a whole, consensus is lacking on general vaccination efficacy and timing to achieve immunogenicity, including holding and repeating vaccines (41–43). Cancer patients were excluded from initial trials for COVID-19 vaccinations, and data on the immunogenicity and safety of COVID-19 vaccines in cancer patients lags behind general pediatrics data. Subsequent studies have explored vaccine safety and efficacy in adult cancer patients at various disease stages of disease (active, remission, post-transplant) (44–51), yet data in pediatric cancer populations remains scarce. We encourage clinicians to acknowledge this lack of data and empathize with caregiver frustrations, affirming their feelings, prior to sharing available information.

Fortunately, healthcare providers can influence decision-making (18, 52). Specifically, caregivers of childhood cancer patients who received information from cancer care professionals were more likely to vaccinate both themselves and their children (27). Although each family is unique, there are common drivers for vaccine decision-making that can be addressed with intention and specificity (1). After asking questions, affirming emotions, and developing therapeutic alliance with caregivers, we advocate for healthcare providers to focus on explaining safety and effectiveness, providing information on logistics for administration, and filling in knowledge gaps in the setting of limited information.

While this analysis focused on the role of healthcare providers in supporting and encouraging families in their decision-making, we also emphasize the critical role that caregivers play in supporting decision-making for other families. In this study, no free-text responses focused on the role of peers or support groups in their own decision-making; however, prior studies have emphasized the value of peers as a form of emotional and informational support in the setting of shared personal experiences in oncology (53). Further research should explore the impact of peer support and guidance in vaccine decision-making.

Finally, caregiver perspectives in this study affirmed themes outlined by the WHO SAGE Vaccine Hesitancy Working Group in their characterization of vaccine hesitancy as “a behavior, influenced by a number of factors including issues of Confidence (do not trust a vaccine or a provider), Complacency (do not perceive a need for a vaccine or do not value the vaccine), and Convenience (access),” also known as the “three Cs” (28). While this study's intent was not to assess vaccine hesitancy, we nevertheless identified themes specific to vaccine decision-making that parallel those raised by individuals who historically expressed hesitancy. Issues related to confidence emerged as discussions of safety or efficacy and concerns regarding the speed in which the vaccine was created with limited information. Complacency materialized across caregiver beliefs that children will not get COVID-19 or will have less severe disease. Convenience manifested in comments specific to logistics, access, and barriers to vaccine availability and administration. Understanding how caregiver perceptions of the COVID-19 vaccine intersect the “three Cs” WHO model can help inform clinical strategies to navigate challenging conversations with families and guide public health messaging. Recent publications have emerged addressing the importance of dynamic public health communication strategies to aid vaccination uptake (54).

This study has several limitations. Certain demographics were not included in survey questions to ensure anonymity. As a result, details on participant gender, age, child age, and relationship to the patient with childhood cancer are unknown. These findings represent the perspectives of those who provided narrative responses, comprising 29% of survey respondents; sample bias may influence findings if participants who shared written responses represent outlier perspectives. However, content analysis of narrative responses indicated a bell curve of opinions, suggesting our findings represent a cross-section of caregivers. Notably, with respect to demographic information collected, qualitative respondents had similar demographics compared to those who opted not to provide free-text responses. The survey techniques relied heavily on internet and social media participation, which risks selection bias with respondents not necessarily representative of all caregivers in their respective countries. Further, survey data skewed toward responses from high income settings; this may reflect varying levels of literacy worldwide as well as unavailability of the survey in languages other than English or Spanish. Findings likely represent a subset of opinions, and further investigations in broader languages and low-income countries are needed. Regardless of commonalities across global responses, conversations must be individualized to the setting and situation. Despite known increased risk with SARS-CoV-2 amongst children with cancer (8), disparate global recommendations exist for childhood vaccinations. Each country has its own standards, with vaccine expansion to younger children or vulnerable populations occurring at different times since the advent of SARS-CoV-2 (55). Finally, we do not know the willingness of respondents to vaccinate themselves, which has been shown to influence perspectives on childhood vaccination (23), or their intent in vaccinating their children, all of which may influence their responses.

In summary, this global study examines the perspectives of caregivers of children with cancer on COVID-19 vaccines and provides insights to guide clinicians in counseling families and providing targeted information to support decision-making. It corroborates findings from the general pediatric population worldwide, with safety and effectiveness, logistics, and limitations in information driving questions and concerns around vaccine uptake and therefore important elements of vaccine counseling. These findings reveal the complexity and multidimensionality of perspectives on COVID-19 vaccination and highlight the interrelated nature of themes. This can help with further development of focused survey tools aimed at understanding attitudes to vaccines amongst the pediatric oncology community. We hope these data may contribute to clinical support tools and public health messaging to help healthcare professionals address vaccine hesitancy and refusal in the context of the COVID-19 vaccine and future novel immunizations for pediatric populations. Further research evaluating how caregiver perspectives influence actual vaccine uptake is needed to guide healthcare professionals in targeting efforts toward supporting medically vulnerable children and their families.

## Data availability statement

The original contributions presented in the study are included in the article/Supplementary material, further inquiries can be directed to the corresponding author.

## Ethics statement

Ethical review and approval was not required for this study on human participants in accordance with the local legislation and institutional requirements. The participants provided their written informed consent to participate in this study.

## St. Jude Children's Research Hospital (SJCRH) and International Society of Paediatric Oncology (SIOP) Parent and Carer Advisory Group

Members of the COVID-19 and Childhood Cancer Vaccine Global Parent and Carer Advisory Group as below contributed to the concept for this study and to the evaluation of its findings:

- Brian Regan, Dana Farber/Boston Children's Pediatric Patient/Family Advisory Committee, United States
- Meghan Shea, Dana Farber/Boston Children's Pediatric Patient/Family Advisory Committee, United States
- Julie Chessell, AC20RN, Canada
- Kim Buff, Momcology, United States
- Carmen Auste, Childhood Cancer International, Philippines
- Pinta Manullang-Panggabean, Yayasan Anyo Indonesia, Indonesia
- Poonam Bagai, Can Kids India, India
- John Ahenkorah, Ghana Parents Group, Ghana

## Author contributions

JB and JG worked to conceptualize and complete data collection for the study. EK led development of the research methodology. JG and AS led the formal analysis and investigation of data. AS wrote the original draft of the paper supervised by EK. All authors revised the paper critically and approved the final version.
