# Supplementary material for: Global caregiver perspectives on COVID-19 immunization in childhood cancer: A qualitative study
Source: Front Public Health. 2023 Mar 7;11:1004263. doi: 10.3389/fpubh.2023.1004263 (PMC10027752; doi:10.3389/fpubh.2023.1004263)
Supplement: Supplementary file 1 [file Table_1.docx]

# Supplemental Table 1. COVID-19 Immunization Survey Instrument.

| **Informed Consent**  Parents and primary caregivers with direct experience of childhood cancer are eligible to complete this survey. No identifiable patient information will be collected.   1. **Do you give your consent for participation in this survey?**   Yes  No |
| --- |
| Which country do you live in?[Drop down list] |
| Which childhood cancer diagnosis do you have experience of? Leukaemia (such as ALL, AML)  Lymphoma (such as B-NHL, Hodgkin’s Disease)  Brain or Spinal tumour (such as Ependymoma, Medulloblastoma)  Solid tumour outside the brain (such as Wilms, Neuroblastoma, Sarcoma)  Other [open ended question] |
| 1. **When did your experience of childhood cancer start?**  Within last 12 monthsBetween 1 and 3 years agoBetween 3 and 5 years agoMore than 5 years ago |
| Section 1: General questions about the COVID-19 vaccines.Please rate the importance of the following questions about the COVID-19 vaccine in children with cancer on a rating scale from Extremely Important (1) to Not Very Important (5).What is the cost of the vaccine?How many vaccine doses are required?Which brand of the vaccine (eg Pfizer, Moderna, Astra Zeneca) should be given?What are the results of vaccine studies in adults?How long will the protection from the vaccine last?What are the benefits of receiving the vaccine?What is the likelihood of a reaction to the vaccine?How well does the vaccine work?Is the vaccine safe? |
| Section 2: General questions about the vaccines and treatment for childhood cancer.Please rate the importance of the following questions about the COVID-19 vaccine in children with cancer on a rating scale from Extremely Important (1) to Not Very Important (5).  Should children with cancer who have had COVID-19 still receive the vaccine?Should children who are on steroids receive the vaccine?Should carers/parents of a child with cancer receive the vaccine?Will the vaccine affect the blood count?Should children who have had a bone marrow transplant receive the vaccine?Should children who have had a previous reaction to a vaccine ingredient still receive the vaccine?Does chemotherapy need to be stopped for the vaccination?Should the vaccine be given on treatment or after all treatment has finished?Does the vaccine interact with chemotherapy?Are children with cancer more likely to have severe side-effects from the vaccine? |
| Please write below any additional questions or comments you have about the COVID-19 vaccine in children with cancer. [open ended question] |
