# Supplementary material for: Global caregiver perspectives on COVID-19 immunization in childhood cancer: A qualitative study
Source: Front Public Health. 2023 Mar 7;11:1004263. doi: 10.3389/fpubh.2023.1004263 (PMC10027752; doi:10.3389/fpubh.2023.1004263)
Supplement: Supplementary file 2 [file Table_2.docx]

**Supplemental Table 2.** CORE-Q (COnsolidated criteria for REporting Qualitative research) Checklist

| Item No. | Topic | Guide Questions/Description | Reported on Page No. |
| --- | --- | --- | --- |
| **Domain 1: Research team and reflexivity** | | | |
| *Personal characteristics* | | | |
| 1 | Interviewer/facilitator | Which author/s conducted the interview or focus group? *No data from interviews or focus groups are presented in this manuscript.* | n/a |
| 2 | Credentials | What were the researcher’s credentials? *Although this manuscript does not present interviewer-facilitated data, we describe the credentials of the researchers who participated in this study.* | Supplemental Table 2 |
| 3 | Occupation | What was their occupation at the time of the study? *Similar as above, we describe the occupations of the researchers on the study team.* | Page 4,  Supplemental Table 2 |
| 4 | Gender | Was the researcher male or female? *We describe the genders of the researchers on the study team and provide initials for all researchers who participated in each stage of the study to enable readers to connect individuals with names within the authorship.* | Pages 4,  Supplemental Table 2 |
| 5 | Experience and training | What experience or training did the researcher have? *We describe the experience and training of the researchers on the study team.* | Supplemental Table 2 |
| *Relationship with participants* | | | |
| 6 | Relationship established | Was a relationship established prior to study commencement? *No data from interviews or focus groups are presented in this manuscript, so there is no interviewer-interviewee relationship to query.* | n/a |
| 7 | Participant knowledge of the interviewer | What did the participants know about the researcher? (e.g., personal goals, reasons for doing the research) *See above. Researchers did not engage with participants with respect to audio-recording of disease reevaluation conversations or completion of surveys.* | n/a |
| 8 | Interviewer characteristics | What characteristics were reported about the interviewer/facilitator? e.g., bias, assumptions, reasons and interest in the research topic? *See above.* | n/a |
| **Domain 2: Study design** | | | |
| *Theoretical framework* | | | |
| 9 | Methodological orientation and theory | What methodological orientation was stated to underpin the study? e.g., grounded theory, discourse analysis, ethnography, phenomenology, content analysis. *Content analysis was used in this study.* | Pages 4-5 |
| *Participant selection* | | | |
| 10 | Sampling | How were participants selected? e.g., purposive, convenience, consecutive, snowball. *We used a convenience sample.* | Page 4 |
| 11 | Method of approach | How were participants approached? e.g., face-to-face, telephone, mail, email. *Participants were mainly recruited via social media, online forums, and email distribution. A small proportion were approached with paper forms.* | Page 4 |
| 12 | Sample size | How many participants were in the study? *There was a total of 627 survey participants with 184 qualitative respondents.* | Page 5, Table 2 |
| 13 | Non-participation | How many people refused to participate or dropped out? Reasons? *Survey was voluntary and data related to refusal to participate or drop out is unknown.* | n/a |
| *Setting* | | | |
| 14 | Setting of data collection | Where was the data collected? e.g., home, clinic, workplace. *Data collected was mainly recruited via social media, online forums, and email distribution. A small proportion were approached with paper forms in a clinic space.* | Page 6-7 |
| 15 | Presence of non-participants | Was anyone else present besides the participants and researchers? *Survey participants were not supervised while completing the survey, unknown if any non-participants may have been present.* | n/a |
| 16 | Description of sample | What are the important characteristics of the sample? e.g., demographic data, date. *Demographic data for participants are described on Page 9 and presented in Table 4.* | Page 8, Table 2 |
| *Data collection* | | | |
| 17 | Interview guide | Were questions, prompts, guides provided by the authors? Was it pilot tested? *There were no interviews carried out in this study, however survey questions were designed via iterative stages of feedback and underwent pilot testing.* | Page 4 |
| 18 | Repeat interviews | Were repeat interviews carried out? If yes, how many? *There were no interviews carried out in this study.* | n/a |
| 19 | Audio/visual recording | Did the research use audio or visual recording to collect the data? *n/a* | n/a |
| 20 | Field notes | Were field notes made during and/or after the interview or focus group? *n/a* | n/a |
| 21 | Duration | What was the duration of the interview or focus group? *n/a* | n/a |
| 22 | Data saturation | Was data saturation discussed? *They survey was open to all eligible caregivers and analysis was not conducted until after the survey was closed. In this context, saturation was not relevant in terms of stopping data collection. However, we describe how saturation was achieved with respect to coding processes.* | Page 4-5 |
| 23 | Transcripts returned | Were transcripts returned to participants for comment and/or correction? *n/a* | n/a |
| *Data analysis* | | | |
| 24 | Number of data coders | How many data coders coded the data? *We describe the number and role and identification of all data coders who participated in this study.* | Page 4,  Supplemental Table 2 |
| 25 | Description of the coding tree | Did authors provide a description of the coding tree or codebook? *We present the codebook in Table 1.* | Table 1 |
| 26 | Derivation of themes | Were themes identified in advance or derived from the data? *Themes were inductively derived from raw data.* | Page 4-5 |
| 27 | Software | What software, if applicable, was used to manage data? *We used MAXQDA software.* | Page 5 |
| 28 | Participant checking | Did participants provide feedback on the findings? *We did not consent patients/caregivers to provide feedback on findings. We did share a draft of the manuscript with the Parent/Carer Advisory Group as a surrogate of participants to provide feedback.* | n/a |
| *Reporting* | | | |
| 29 | Quotations presented | Were participant quotations presented to illustrate the themes/findings? Was each quotation identified? e.g., participant number. *Representative quotes are embedded within the text, with additional quotes presented in the comprehensive Table 3.* | Pages 5-6, Table 3 |
| 30 | Data and findings consistent | Was there consistency between the data presented and the findings? *We demonstrate consistency between data presented in the Results section and interpretation of findings delineated in the Discussion section.* | Pages 5-9 |
| 31 | Clarity of major themes | Were major themes clearly presented in the findings? *We presented all major themes in detail.* | Pages 5-9, Figure 1 |
| 32 | Clarity of minor themes | Is there a description of diverse cases or discussion of minor themes? *We provide a variety of rich quotes in a comprehensive table to offer readers diversity of cases.* | Table 3 |

Developed from: Tong A, Sainsbury P, Craig J. Consolidated criteria for reporting qualitative research (COREQ): a 32-item checklist for interviews and focus groups. *International Journal for Quality in Health Care*. 2007. Volume 19, Number 6: pp. 349 – 357.
