# Supplementary material for: Global caregiver perspectives on COVID-19 immunization in childhood cancer: A qualitative study
Source: Front Public Health. 2023 Mar 7;11:1004263. doi: 10.3389/fpubh.2023.1004263 (PMC10027752; doi:10.3389/fpubh.2023.1004263)
Supplement: Supplementary file 3 [file Table_3.docx]

**Supplemental Table 3.** Research Team Attributes and Qualifications

| **Author** | Attributes and Qualifications |
| --- | --- |
| A.S. | Female, white physician with a Medical Degree, a Master’s in Genomic Medicine, qualitative research training, global health training, and clinical training and practice in pediatrics and hospice and palliative medicine. |
| J.G. | Female, white social-scientist with a Doctorate degree in Quantitative Sociology, a Master’s in Social Policy Analysis, graduate-level training in Economics, mother and carer of a child with Anaplastic Large Cell Lymphoma. |
| J.B. | Female, British Sri Lankan with a Medical Degree and Doctorate degree, clinical training and practice in pediatric hematology-oncology |
| E.K. | Female, white physician-scientist with a Medical Degree, a Master’s in Public Health, graduate-level training in qualitative research methodology with a focus on communication science, and clinical training and practice in pediatric hematology-oncology and hospice and palliative medicine. |
